# Potential Activities of *Centella asiatica* Leaf Extract against Pathogenic Bacteria-Associated Biofilms and Its Anti-Inflammatory Effects

**DOI:** 10.1155/2024/5959077

**Published:** 2024-09-09

**Authors:** Piriya Chonsut, Chonticha Romyasamit, Atthaphon Konyanee, Nattisa Niyomtham, Lavanya Goodla, Auemphon Mordmuang

**Affiliations:** ^1^ Department of Applied Thai Traditional Medicine School of Medicine Walailak University, Nakhon Si Thammarat 80160, Thailand; ^2^ Department of Medical Technology School of Allied Health Sciences Walailak University, Nakhon Si Thammarat 80160, Thailand; ^3^ Department of Medical Sciences School of Medicine Walailak University, Nakhon Si Thammarat 80160, Thailand; ^4^ International College of Dentistry Walailak University, Bangkok 10400, Thailand; ^5^ Department of Biochemistry and Molecular Biology University of New Mexico School of Medicine, Albuquerque 87131, NM, USA

## Abstract

The medicinal value of *Centella asiatica* leaf extract was evaluated as an alternative treatment. The chemical composition of the leaf extract was analyzed, and the biological activities were determined. High-performance liquid chromatography coupled with a photodiode array detector (HPLC-PDA) was used to identify the asiatic acid, madasiatic acid, and madecassic acid/Brahmic acid isolated from the ethanolic extract. The plant extract at 25 mg/disk was found to inhibit both Gram-positive and Gram-negative pathogenic bacteria by the agar disk diffusion test. The MIC and MBC of the ethanolic extracts were better than those of the aqueous extracts. The ethanolic extracts showed antibacterial activity against Gram-positive bacteria with MICs and MBCs ranging from 1.024 to 2.048 mg/mL and 2.048 to 4.096 mg/mL, respectively. The remarkable antibacterial activities were observed against *S. mutans*. The ethanolic extract at a concentration of 1/2 × MIC exhibited the inhibition effect on *S. mutans* biofilm formation like the activity of 0.2% chlorhexidine and significantly modified hydrophobicity of the bacterial cell surface. The effects were confirmed via molecular docking analysis. The binding affinities of asiatic acid, madecassic acid, and madasiatic acid with glucosyltransferase C (GtfC) of *S. mutans* exhibited superior strength in comparison with alpha-acarbose and chlorhexidine. Moreover, the nitric oxide (NO) secretion of RAW247.6 cells was determined after treating the cells with concentrations of the extract. The *C. asiatica* ethanolic extract can inhibit the secretion of NO, which can inhibit the inflammatory process. The findings indicate the applications of the *C. asiatica* ethanolic extract as the alternative anti-*S. mutans* agent and could be used for further formulation for the treatment and prevention of dental diseases and inflammatory injury in the oral cavity.

## 1. Introduction

*Centella asiatica*, commonly known as Gotu kola, boasts a rich historical significance intertwined with various cultures and traditions. Its roots in traditional medicine stretch back centuries, with ancient texts from Ayurveda, traditional Chinese medicine (TCM), and traditional African medicine all lauding its therapeutic properties. In Ayurveda, this herbal plant has been revered as a “Medhya Rasayana,” an herb that promotes mental clarity and cognitive function. It has been used to enhance memory, improve concentration, and alleviate anxiety and stress. An aqueous leaf extract of the plant has been shown to increase synaptic density, improve cognitive function, and demonstrate promising effects on memory retention in rats. Researchers attributed these effects to the herb's ability to enhance the expression of the antioxidant response gene NRF2 [1]. Moreover*, C. asiatica* has garnered attention for its neuroprotective effects, with research suggesting its potential to mitigate age-related cognitive decline and neurodegenerative disorders, such as Alzheimer's disease [2]. Gray et al. investigated the neuroprotective potential of *C. asiatica* in an animal model of Alzheimer's disease. The findings suggested that the herb's anti-inflammatory and antioxidant properties may help protect against neurodegeneration and cognitive decline [3]. In TCM, *C. asiatica* is valued for its ability to cool inflammation, promote wound healing, and support the health of the skin. It is often utilized to treat conditions, such as eczema, psoriasis, and various inflammatory skin disorders. Somboonwong et al. demonstrated the anti-inflammatory effects of the plant extract and its ability to accelerate wound healing. The study suggested that the herb promotes collagen synthesis and angiogenesis, contributing to faster tissue repair [4]. Furthermore, emerging research suggests that *C. asiatica* may have benefits in managing conditions, such as diabetes, cardiovascular diseases, and gastrointestinal disorder, although further clinical studies are warranted to elucidate its full therapeutic potential in these areas. The effects of the plant extract on cardiovascular health and metabolic disorders were explored. The study indicated that *C. asiatica* supplementation may have beneficial effects on lipid metabolism, blood pressure regulation, and insulin sensitivity [5].

The traditional use of *C. asiatica* has gained scientific interest due to its diverse bioactive components, such as triterpenoids, flavonoids, and asiaticoside [6, 7]. These compounds have been extensively studied for their pharmacological activities, including anti-inflammatory, antioxidant, and neuroprotective effects [2, 6]. These properties provide a scientific basis for the traditional use of *C. asiatica* across different medical conditions.

Several studies have reported the antibacterial potential of *C. asiatica* extracts and their bioactive compounds. Research conducted by Pitinidhipat et al. demonstrated the inhibitory effects of *C*. *asiatica* against both Gram-positive and Gram-negative bacteria [8]. The essential oil extracts from *C. asiatica* demonstrated the activity to inhibit *Bacillus subtilis, Staphylococcus aureus*, *Escherichia coli*, *Pseudomonas aeruginosa*, and *Shigella sonnei* [9]. The study highlighted the presence of bioactive compounds, such as triterpenoids and flavonoids, which contribute to the antibacterial activity [10, 11]. This finding suggests that the plant extract may have the potential to serve as a natural alternative to conventional antibiotics. It has a high content of antioxidant compounds, including asiaticoside and madecassoside [12, 13]. A study by Chintapanti et al. explored the antioxidant properties of *C. asiatica* in an animal model, demonstrating its ability to enhance antioxidant enzymes and decrease oxidative stress markers [14]. These findings indicated that the plant may have a role in mitigating oxidative damage associated with various diseases including cell injuries caused by the infections.

Moreover, inflammation underlies many chronic diseases, making the search for natural anti-inflammatory agents crucial. A study by Dong et al. investigated the anti-inflammatory effects of *C. asiatica* in a mouse model of inflammation-induced lung injury. The researchers found that treatment with the herbal extract significantly reduced inflammation mediators and improved lung function [15]. The mentioned studies highlight the bioactive compounds present in *C. asiatica* and their pharmacological activities, offering insights into its potential therapeutic applications. However, further research is required to elucidate the mechanisms of action and optimize the usage of *C. asiatica* in clinical practice. Understanding the efficiency of *C. asiatica* in various medical applications could not only contribute to the development of new natural remedies but also help in the discovery of novel bioactive compounds for pharmaceutical applications. Hence, this research article aims to consolidate the current knowledge and encourage further investigations into the vast potential of *C. asiatica* as a valuable medicinal plant.

## 2. Materials and Methods

### 2.1. Materials

Acetonitrile and methanol (HPLC grade) were purchased from RCI Labscan (Bangkok, Thailand). Deionized water (18.2 MΩ-cm) was purified using a Milli-Q system (Millipore, Billerica, MA, USA). Ethanol and formic acid (analytical grade) were purchased from Merck (Darmstadt, Germany).

### 2.2. Plant Materials and Preparation of Herbal Extracts

Fresh *Centella asiatica* leaves were collected in 2021 from Chian Yai Subdistrict and Mae Chao Yu Hua Subdistrict, Nakhon Si Thammarat Province, Thailand. Herbarium voucher specimens were *Centella asiatica* SM 0324030901. They were deposited at Applied Thai Traditional Medicine, School of Medicine, Walailak University, Nakhon Si Thammarat, Thailand. The plant materials were dried using a hot air oven at 60°C for 72 hours. All dried herbs were ground into coarse powders. To obtain crude ethanolic extracts, pulverized herbs were macerated in 95% ethanol (1 : 10 w/v) for 7 days. For aqueous extracts, the herbs were subjected to 6-hour cycles of Soxhlet extraction. Four repeated cycles were performed using a sample-to-solvent ratio (g/mL) of 1 : 10 at the temperature of 45° ± 2°C. The macerates were then filtered and dried using a rotary evaporator. The crude extracts were stored at −20°C until further use. The plant extracts were dissolved in dimethyl sulfoxide (DMSO) and diluted to obtain a final concentration of the solvent less than 10% before use for antibacterial assay and antibiofilm formation testing.

### 2.3. Qualitative Phytochemical Analysis of Herbal Extracts

The herbal extract and triterpene standards were prepared at 1.0 mg/mL in methanol and filtered through a 0.45 *μ*m nylon membrane syringe filter and subjected to high-performance liquid chromatography coupled with a photodiode array detector (HPLC-PDA). The analysis was carried out with Shimadzu Nexera LC-40 series with a photodiode array detector and autosampler (Shimadzu, Kyoto, Japan). Separation was achieved at 30°C on a 250 mm × 4.6 mm, 5 *μ*m (Tosoh Bioscience, Tokyo, Japan). The detection wavelength was set at 210 nm. The separation was done by a gradient elution program of water (solvent A) and acetonitrile (solvent B) at a flow rate of 1.2 ml/min. The gradient system was performed as follows: 0–10 min, 15–80% B; 10–15 min, 80% B; and 15–20 min, 80−15% B. The injection volume was 5 *μ*l.

### 2.4. Bacterial Culture and Conditions

In this study, seven strains of pathogenic bacteria known for their ability to form biofilms were used, including *Staphylococcus aureus* ATCC 25923*, S. epidermidis* ATCC 35984*, Streptococcus mutans* ATCC 25175*, Escherichia coli* ATCC 25922*, Klebsiella pneumoniae* ATCC 700603*, Acinetobacter baumannii* ATCC 17978, and *Pseudomonas aeruginosa* ATCC 27853. The bacteria were cultured overnight in brain heart infusion (BHI) broth. The culture was adjusted to the turbidity of a 0.5 McFarland standard or a measured optical density (OD) of 0.1 (approximately 1 × 10^8^ CFU/mL) at 600 nm for antibacterial testing. The bacterial suspensions were diluted 1 : 100 in 1 mL of Tryptic Soy Broth (TSB) containing 2% sucrose (Merck, Darmstadt, Germany) and were then transferred into sterile round-bottom 96-well polystyrene microplates (SPL Life Sciences Co., Korea). The bacterial biofilm was grown at 37°C for 24 hours to test antibacterial biofilm formation.

### 2.5. Agar Disk Diffusion Assay

The antimicrobial activity of the aqueous and ethanolic extracts was determined by the agar disk diffusion method [16] The broth culture of the bacterial strain was adjusted to the density of 0.5 McFarland standard. An aliquot of 0.1 mL of the bacterial suspension was spread on cation-adjusted Mueller–Hinton agar (CAMHA) plates. The 6-mm-diameter paper disks loaded with 25 mg of extract were placed on the media and incubated at 37°C overnight. Zones of growth inhibition were measured in millimeters. Standard disks of 5 *μ*g ciprofloxacin and 30 *μ*g vancomycin were used as a positive control, while a disk of 1% DMSO was used as a negative control. All tests were performed in triplicate, and the mean values of the diameter of the inhibition zone ± standard deviation were determined after incubation at 37°C for 24 hours.

### 2.6. Evaluation of Minimum Inhibitory Concentration (MIC) and Minimum Bactericidal Concentration (MBC)

The broth microdilution method determined the MICs and MBCs of the plant extract according to the Clinical and Laboratory Standards Institute (CLSI) guidelines (2018). A 20 *μ*L aliquot of the ethanolic extract and antibiotics was separately added into a 96-well microtiter plate and diluted by performing twofold serial dilution. The total volume was made up to 100 *μ*L by adding 80 *μ*L of Mueller–Hinton broth (MHB) into each well. 100 *μ*L of bacterial suspension (10^6^ CFU/mL) was inoculated in the wells and incubated at 37°C for 18 hours. Ciprofloxacin and vancomycin were used as positive controls, while MHB media and solution of 1% DMSO served as a growth and negative control, respectively. To determine the MICs, the absorbance of the cultures and controls was measured at 600 nm using a microplate reader (BioTek Instruments, Inc., USA). All tests were performed in triplicate. The value of MBC was subsequently estimated by streaking aliquots of the MIC on BHI agar and then cultured at 37°C for 18–24 hours. The plates were observed for bacterial growth.

### 2.7. Inhibition of Bacterial Biofilm Formation Assay

The different concentrations of *C*. *asiatica* extract at sub-MICs and the MIC were subjected to the bacterial culture and grown at 37°C for 24 hours. After incubation, the culture wells were gently washed twice with sterile phosphate-buffered saline (PBS, pH 7.3) and air-dried. An aliquot of 200 *μ*L of 0.1% crystal violet solution (Merck, Darmstadt, Germany) was added into each well to stain the bacterial biofilm for 15 min at room temperature. Any excess stain was removed by rinsing with distilled water and allowed to dry. The biomass of bacterial biofilm in the wells was determined by decolorization with 200 *μ*L of 33% acetic acid for 15 min and evaluated using a microtiter plate reader (BioTek Instruments, Inc., USA) at 570 nm. A final concentration of 0.2% w/v of chlorhexidine gluconate in water solution was used as a standard drug, and 1% DMSO was used as a negative control. The experiment was performed in triplication. The percentage of inhibition was calculated by comparing the intensity of the biofilm biomass in the negative control using the equation: [(OD control-OD treatment)/OD control] × 100.

### 2.8. Microbial Adhesion to Hydrocarbon (MATH) Test

MATH assay was performed to evaluate the bacterial cell surface hydrophobicity. *S. mutans* ATCC 25175 was used as a representative of biofilm-producing bacteria in this assay. The bacteria were cultured in BHI broth containing the plant ethanolic extract with the final concentration of 1/4 × MIC to 4 × MIC, at 37°C for 4 hours. The cell pellets were collected by centrifugation at 4000 × g for 5 min and washed twice with sterile saline solution. The cell density was adjusted to an OD of 0.3 at 600 nm (OD initial). The bacterial cells incubated without the extract were used as a control. Three milliliters of the cell suspension were put into a glass tube adding 0.25 mL of toluene reagent. The tubes were thoroughly mixed for 2–3 minutes using a vortex machine and kept at room temperature for 10 min. After the toluene phase had separated from the culture phase, the OD of the aqueous phase (OD final) was determined at 600 nm by spectrophotometry. The bacteria with a hydrophobic index greater than 60% were classified as hydrophobic. The tests were performed in triplicate. The hydrophobicity index (%) was calculated as follows:

(1) $$\begin{array}{c} \left[\frac{\left(O.D.\text{ initial }-\;O.D.\text{ final}\right)}{O.D.\text{ initial}}\right]\times 100. \end{array}$$

### 2.9. Molecular Docking Study

The three-dimensional (3D) crystal protein structure of the glucansucrase, also known as glucosyltransferase C (GtfC), from *S. mutans* [17] [Protein Data Bank (PDB) ID: 3AIC] with a resolution of 3.11 Å was obtained from the Research Collaboratory for Structural Bioinformatics (RCSB) PDB (https://www.rcsb.org) in PDB format. The chemical structures of asiatic acid (PubChem CID: 119034), madecassic acid (PubChem CID: 73412), madasiatic acid (PubChem CID: 23132225), and chlorhexidine (PubChem CID: 9552079) were retrieved from the PubChem database (https://pubchem.ncbi.nlm.nih.gov, accessed on June 16, 2023) in a simple data format (SDF). These structures were subjected to geometry optimization and energy minimization using the Merck molecular force field (MMFF94s) [18, 19] in the Avogadro software version 1.2.0. Gasteiger charges were added, and nonpolar hydrogen atoms were merged using AutoDockTools v. 4.2.6. The resulting ligand structures were saved in the protein data bank, partial charge, and atom type (PDBQT) format. AutoDockTools was used for the preparation of the protein structure, which involved the removal of cocrystallized ligands (alpha-acarbose and 2-(n-morpholino)-ethanesulfonic acid), the removal of water molecules, the addition of polar hydrogens, and the assignment of Kollman charges. The active site of GtfC, including Glu515, Asp477, Asp588, Arg475, His587, Tyr916, Tyr430, Leu433, Asn481, and Trp517, was determined based on the amino acids interacting with alpha-acarbose present in the protein. A grid box was created to cover these regions using AutoDockTools, with dimensions of 24 × 24 × 24 Å^3^ for *x*,  *y*, and *z* points, a grid spacing of 1.000 Å, and the center coordinates (*x* = 192.011, *y* = 45.529, *z* = 194.613 Å). Molecular docking was performed using AutoDock Vina v. 1.1.2, with an exhaustiveness value of 24 and other parameters set as default. The compounds with the lowest binding energy (kcal/mol) and minimum root mean square deviation (RMSD) were selected as the most suitable docking poses. To validate the docking process, the native ligand (alpha-acarbose) molecule was redocked into the identified active site of GtfC under the same conditions, and the resulting RMSD of the redocked ligand should be below 2.5 Å to confirm the reliability of this method before proceeding with the experiment. The hydrogen bonds and hydrophobic interactions between ligand atoms and amino acid residues of GtfC were identified using the Protein-Ligand Interaction Profiler (PLIP) online web server with default parameters. The protein-ligand complexes were visualized using the PyMOL molecular graphics system v. 2.5.2.

### 2.10. Cell Culture

The RAW 264.7 cells, a type of mouse macrophage cells, were obtained from ATCC. The cells were cultured in Dulbecco's modified Eagle's medium (DMEM; Gibco, Thermo Fisher Scientific, NY, USA) supplemented with 10% fetal bovine serum (Gibco) and 1% penicillin-streptomycin solution (Gibco, Thermo Fisher Scientific) at 37°C in 5% CO_2_. The RAW 264.7 cells were subcultured and plated when they reached 80% to 90% confluency.

### 2.11. Cell Viability Assays

The toxicity of the *C. asiatica* ethanoic extract was assessed in RAW 264.7 cells using a 3-(4,5-dimethylthiazol-2-yl)-2,5-diphenyl tetrazolium bromide (MTT) assay. Briefly, RAW 264.7 cells were seeded onto 96-well microplates at 1 × 10^5^ cells/mL and incubated at 37°C in a 5% CO_2_ incubator for cytotoxicity assays. The ethanolic extracts were added to plates after dilutions with final concentrations ranging from 0.16–10,000 *μ*g/ml and incubated at 37°C for 24 h. After incubation, supernatants were discarded, and the cells were washed with PBS. A volume of 50 *μ*L of 3-(4,5-dimethylthiazol-2-yl)-2,5-diphenyl tetrazolium bromide (MTT) solution (Sigma, MO, USA) (0.5 mg/mL in DMEM) was added to each well and incubated for 4 h in the dark after removing the treatment mixture from each well. The formazan crystals were dissolved by adding 200 *μ*L of dimethylsulfoxide (DMSO) solution (Sigma, MO, USA). The OD was measured at 570 nm using a microplate reader. The median lethal concentration (LC50) of substances was calculated by dose-response relationships/sigmoidal curve fitting analysis. Ten percent lethal concentration (LC10) was selected as an appropriate concentration for cellular experiments. The experiment was performed in triplicate.

### 2.12. Nitric Oxide Assays

To assess their potential anti-inflammatory effects, the ability of the ethanolic extracts to decrease the production of nitric oxide (NO) induced by lipopolysaccharide (LPS) in RAW 264.7 cells was examined. The cell suspension was seeded in a 24-well microplate and treated with 72.28 *μ*g/L of the extract with or without 1 *μ*g/mL of LPS obtained from Sigma-Aldrich (St. Louis, USA). Cells treated with 1 *μ*g/mL of LPS alone were used as a positive control. Aspirin was prepared in DMSO before being added to the well as a negative control. The final concentration of DMSO in the medium was 1/1000 (v/v). After a 24-hour incubation at 37°C in 5% CO_2_, the nitric oxide production was quantified by treating the supernatant with an equal volume of Griess reagent (Sigma-Aldrich, St. Louis, USA). The OD was measured at 570 nm using a microplate reader. Each test was performed in triplicate. The concentration of nitric oxide production was calculated using the following equation:

(2) $$\begin{array}{c} \left(\frac{\text{OD of test}}{\text{OD of standard}}\right)\times \text{concentration of standard}. \end{array}$$

### 2.13. Statistical Analysis

Values of each parameter are expressed as the mean ± standard error of the mean (SEM). Comparisons among different groups were performed by one-way analysis of variance (ANOVA). When significant differences existed, Dunnett's multiple-range tests were used to compare the means. A probability of *p* < 0.05 was considered significant.

## 3. Results

### 3.1. Antibacterial Activity of the Ethanolic and Aqueous Extracts of *C. asiatica* Leaves against Pathogenic Bacteria

The plant extracts were preliminarily tested for antibacterial activity by agar disk diffusion method. The results are shown in Table 1. The antibacterial activity of the *C. asiatica* ethanolic extract was noted in the activity of the aqueous extracts. The ethanolic extract exhibited the greater inhibitory effects against the tested Gram-positive strains than Gram-negative strains in this study. The inhibition zones of the ethanolic extract against Gram-positive and Gram-negative bacteria were ranging from 9.6–14.3 mm and 7.3–9.2 mm, respectively. The aqueous extracts showed inhibitory activity against Gram-positive and Gram-negative ranging from 6.5–8.4 mm and 6.2–6.8 mm, respectively. Moreover, the largest inhibitory zone was observed in the effect of *C. asiatica* ethanolic extract against *S. mutans* ATCC 25175, which was 14.3 mm. The disk of vancomycin showed inhibitory activities on Gram-positive bacterial strains ranging from 17.5–24.2 mm. Ciprofloxacin provided the effects against Gram-negative strains ranging from 18.5–21.5 mm.

The MIC and MBC values of the *C. asiatica* extracts are shown in Table 2. The MIC and MBC of the ethanolic extracts were better than those of the aqueous extracts. The ethanolic extracts showed antibacterial activity against Gram-positive bacteria with MICs and MBCs ranging from 1.024 to 2.048 mg/mL and 2.048 to 4.096 mg/mL, respectively, while the aqueous extracts demonstrated MIC values ranging from 16.384 to 32.768 mg/mL, and MBC values were 32.768 to more than 65.536 mg/mL. The plant ethanolic extract and the aqueous extract possessed the activity to inhibit Gram-negative bacterial strains with the ranging of MIC/MBC values of 8.192 to 16.384/16.384 to 32.768 mg/mL and >32.768/>65.536 mg/mL, respectively. The tested bacterial stains were all susceptible to standard antibiotics according to CLSI antibacterial testing standards.

### 3.2. Phytochemical Components of the Ethanolic Extracts of *C. asiatica*

**Figure 1:**
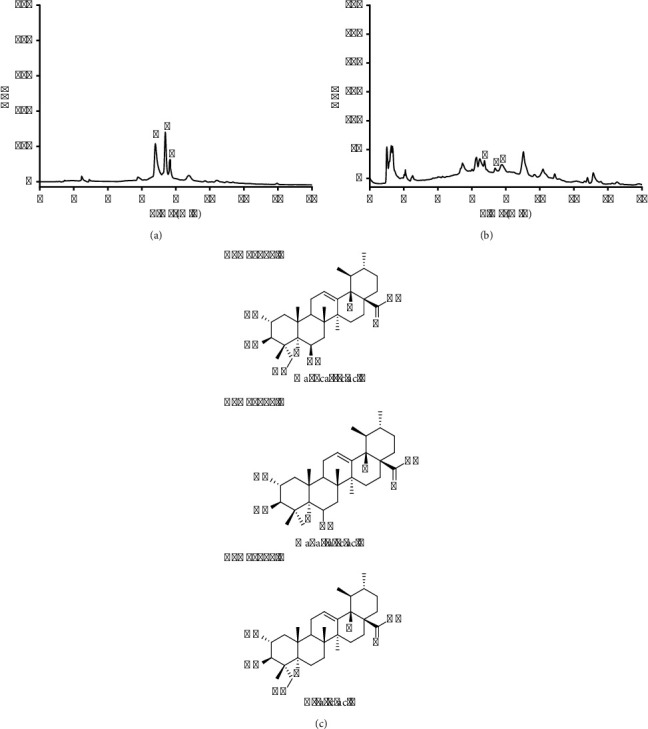
High-performance liquid chromatography coupled with a photodiode array detector (HPLC-PDA) chromatograms of triterpene acid mixed standards (a) and *Centella asiatica* ethanolic extract (b) were obtained, along with the corresponding chemical structures of the isolated compounds (c). The spectra exhibited distinct peaks corresponding to madecassic acid (1), madasiatic acid (2), and asiatic acid (3).

The ethanolic extracts of *C. asiatica* demonstrated stronger antibacterial effects compared to the aqueous extracts. Consequently, a qualitative analysis of the ethanolic extracts was conducted using high-performance liquid chromatography coupled with a photodiode array (HPLC-PDA). The analysis was performed using both negative and positive ionization modes to determine the chemical composition of the extract. Drawing from prior research, we identified candidate compounds in the extract that might be responsible for its antibacterial properties. The proposed negative and positive ions corresponding to various compounds are presented in Table 3. Three principal compounds of madecassic acid, madasiatic acid, and asiatic acid were detected in the ethanolic extracts of *C. asiatica* leaves at specific retention times (RTs). Madecassic acid was observed at an RT of 6.7, madasiatic acid at 7.3, and asiatic acid at 7.7. Furthermore, the HPLC-PDA chromatogram of triterpene acid standards, along with the isolated compounds, is illustrated in Figure 1. The photodiode array detector was used to measure light absorption across a broad range of wavelengths, capturing a spectral profile for each peak of the compounds (Figure 1(b)), which was then compared with the spectra of the reference standards (Figure 1(a)).

### 3.3. Inhibitory Activity of *S. mutans* Biofilm Formation

**Figure 2:**
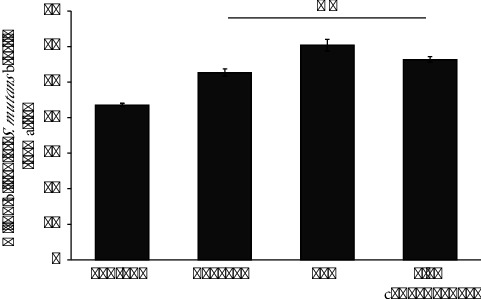
Inhibitory effects of *Centella asiatica* ethanolic extract at the concentration of 1/4 × MIC, 1/2 × MIC, and the MIC on *S. mutans* biofilm formation within 24 hours compared to the activities of 0.2% chlorhexidine standard solution.

The impact of *C. asiatica* ethanolic extracts on inhibiting *S. mutans* ATCC 25175 biofilm production is depicted in Figure 2. Bacterial cells were exposed to various concentrations of the ethanolic extract, specifically at 1/4 × MIC, 1/2 × MIC, and MIC over 24 hours to assess biofilm formation. Biofilm inhibition percentages were determined by comparing biofilm density with a negative control. Results indicate concentration-dependent inhibitory effects of the ethanolic extract. A 0.2% chlorhexidine solution demonstrated 56.30% inhibition of *S. mutans* biofilm formation. Correspondingly, ethanolic extracts at 1/4 × MIC, 1/2 × MIC, and MIC concentrations exhibited biofilm inhibition percentages of 43.50%, 52.62%, and 60.36%, respectively. Notably, the ethanolic extract at 1/2 × MIC and MIC concentrations showed similar inhibitory effects on *S. mutans* biofilm formation compared to 0.2% chlorhexidine, with no statistically significant difference (*p* value >0.05).

### 3.4. Effects of *C. asiatica* Ethanolic Extracts on *S. mutans* Cell Surface Hydrophobicity

**Figure 3:**
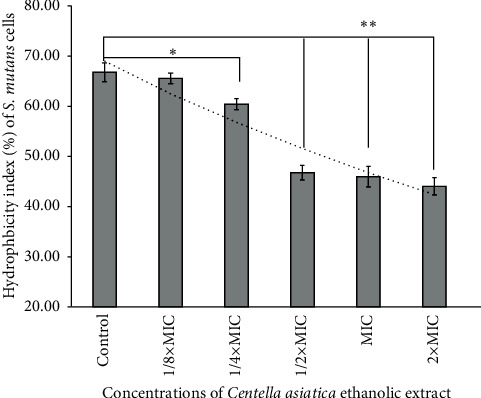
Effects of the *Centella asiatica* ethanolic extracts on *S. mutans* ATCC 25175 cell surface hydrophobicity. The hydrophobicity index was quantified after treating the bacterial cells with 1/8 × MIC, 1/4 × MIC, 1/2 × MIC, 1 × MIC, and 2 × MIC of the extract.

The modification effects of the ethanolic extracts on bacterial cell surface were determined. We hypothesized that the plant extract may modify the cell surface hydrophobic properties, which affects the aggregation activity of the bacteria to the host cells. *S. mutans* ATCC 25175 were selected as the representatives of hyper-biofilm-producing bacterial strains in this experiment. The bacteria were classified as hydrophobic bacteria with a hydrophobicity index greater than 60%. After treating the bacterial cells with the ethanolic extracts of *C. asiatica* at a concentration of 1/8 × MIC, 1/4 × MIC, 1/2 × MIC, and the MIC, the *S. mutans* cells possessed significant lower level of hydrophobicity than the untreated cells. The ethanolic extract modified the bacterial cell surface by decreasing the hydrophobicity in a concentration-dependent manner. The concentration of 1/4 × MIC of the ethanolic extracts could significantly reduce the cell hydrophobicity of *S. mutans*, which is shown in Figure 3. The hydrophobicity of the bacterial cells was significantly decreased to reach an index lower than 50% by exposure to the concentration of 1/2 × MIC of the ethanolic extracts.

### 3.5. Molecular Docking Analysis

The results obtained from the molecular docking analysis in this study revealed the binding affinities of GtfC with several compounds, including alpha-acarbose (a cocrystallized ligand and known inhibitor), chlorhexidine (employed as a positive control), and natural compounds derived from *C. asiatica*, namely, asiatic acid, madecassic acid, and madasiatic acid. The computed binding affinities were determined to be −8.2 kcal/mol, −8.4 kcal/mol, −8.8 kcal/mol, −10.0 kcal/mol, and −10.0 kcal/mol, respectively (Table 4). It is important to note that in the context of molecular docking analysis, higher negative scores correspond to stronger binding affinities between the ligand and the protein [20]. Remarkably, in terms of energy considerations, the binding affinities of asiatic acid, madecassic acid, and madasiatic acid with GtfC exhibited superior strength in comparison with alpha-acarbose (a known inhibitor) and chlorhexidine (a positive control) when interacting with GtfC.

**Figure 4:**
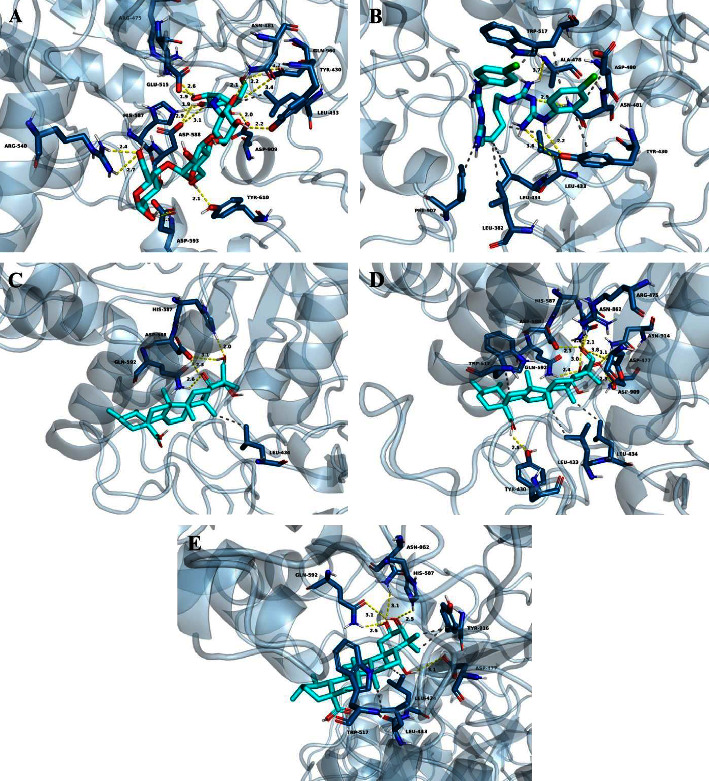
Docking poses of alpha-acarbose (a cocrystallized ligand and known inhibitor) (a), chlorhexidine (a positive control) (b), asiatic acid (c), madecassic acid (d), and madasiatic acid (e) in the active site of GtfC of *S. mutans* (PDB: 3AIC). Hydrogen bonding interactions were visualized with yellow dotted line, while hydrophobic interactions were represented by gray dotted lines.

The molecular interactions between the ligands and amino acid residues of GtfC were examined. Alpha-acarbose formed hydrogen bonds with amino acid residues, such as Tyr430, Arg475, Asn481, Glu515, Arg540, His587, Asp588, Asp593, Tyr610, Asp909, and Gln960, while also establishing hydrophobic interactions with Leu433 (Figure 4(a)). Similarly, chlorhexidine formed hydrogen bonds with Tyr430, Ala478, and Asn481 and established hydrophobic interactions with Leu382, Tyr430, Leu433, Leu434, Asp480, Trp517, and Phe907 (Figure 4(b)).

Regarding the natural compounds, it was observed that asiatic acid formed hydrogen bonds with His587, Asp588, and Gln592 and established hydrophobic interactions with Leu434 (Figure 4(c)). Madecassic acid exhibited hydrogen bonding with Tyr430, Arg475, Asp477, His587, Asp588, Gln592, Asn862, Asp909, and Asn914, while establishing hydrophobic interactions with Leu433, Leu434, and Trp517 (Figure 4(d)). Madasiatic acid formed hydrogen bonds with Asp477, His587, Gln592, and Asn862 and established hydrophobic interactions with Leu433, Leu434, Trp517, and Tyr916 (Figure 4(e)).

### 3.6. Cell Viability and Cytotoxicity

Cytotoxicity of the ethanolic *C. asiatica* extracts in RAW 264.7 cells was performed using MTT assays. The dose-response/sigmoidal curve fitting analysis of percent cell viability was established. LC50 and LC10 of ethanolic *C. asiatica* extracts were 229.61 *μ*g/mL and 72.28 *μ*g/mL, respectively. Thus, the ethanolic *C. asiatica* extracts were considered safe for further evaluation.

### 3.7. NO Production

**Figure 5:**
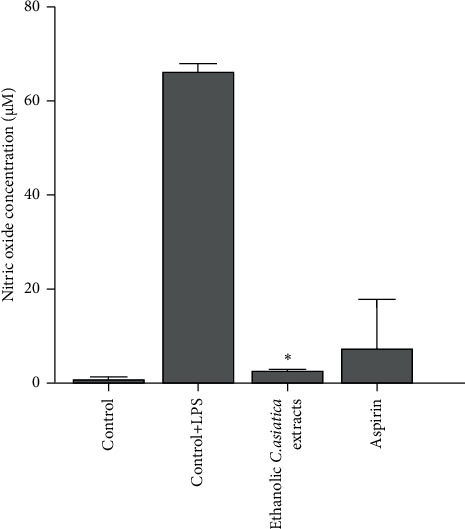
Inhibition of nitric oxide production in the LPS-stimulated RAW264.7 cells treated with the ethanolic *C. asiatica* extracts and aspirin as a control. ^∗^The statistical significance (*p* value <0.05).

NO is a versatile signaling molecule that plays a crucial role in the immune response to inflammation. Results of the NO assay (Figure 5) established that ethanolic *C. asiatica* extracts reduced the NO production to 2.66 ± 0.27 M in LPS-stimulated RAW 264.7 cells when compared with untreated LPS-stimulated RAW 264.7 cells (66.15 ± 1.75 *μ*M). The NO production of aspirin-treated LPS-stimulated RAW 264.7 cells was 7.34 ± 10.44 *μ*M and was not significantly different from that of the ethanolic *C. asiatica* extracts treated LPS-stimulated RAW 264.7 cells.

## 4. Discussion

*C. asiatica* has been revered for its medicinal properties in traditional medicine systems for centuries. It originates from Southeast Asia [6, 7] and belongs to the Apiaceae family. Scientific research has begun to validate many of these traditional uses, shedding light on the diverse medicinal benefits of this plant [21–23]. One of the notable medicinal benefits of *C. asiatica* is its potential as an anti-inflammatory agent. Studies have shown that it can help reduce inflammation in the body and alleviate symptoms associated with conditions [15, 24]. *C. asiatica* also exhibits antioxidant properties, which can help protect the body against oxidative stress and damage caused by harmful free radicals [6]. Furthermore, *C. asiatica* has demonstrated abilities to support the nervous system. It is believed to have a positive impact on cognitive function, memory, and mental clarity [2]. Some studies have suggested that it may even have potential in the management of neurological disorders, such as Alzheimer's disease. Additionally, the plant has shown promise in its antibacterial properties. It has been found to inhibit the growth of various harmful bacteria, including strains that are resistant to antibiotics [8, 25–27]. This makes it a potentially valuable natural alternative for combating bacterial infections. Moreover, it has been associated with potential benefits for wound healing and scar reduction. It is believed to stimulate collagen production and enhance skin cell regeneration, aiding in the recovery of wounds and preventing excessive scarring. It is important to note that while *C. asiatica* shows promising medicinal benefits, further research is still needed to fully understand its mechanisms of action and confirm its efficacy. As with any herbal remedy, it is recommended to consult with a healthcare professional before incorporating *C. asiatica* into your medical treatment.

The ethanolic extracts exhibited superior antibacterial activity in comparison with the aqueous extracts. This was evidenced by the lower MIC and MBC values observed for the ethanolic extract. These findings indicate the efficacy of the ethanolic extract at lower concentrations, suggesting its potential as a powerful antibacterial agent. In contrast, the aqueous extracts demonstrated higher MIC and MBC values, implying that higher concentrations are necessary to achieve similar antibacterial effects. This emphasizes the significance of the solvent used in the extraction process, specifically in the context of the agar disk diffusion method.

Furthermore, our investigation revealed that the *C. asiatica* ethanolic extract displayed notable inhibitory effects primarily against Gram-positive strains, whereas its impact on Gram-negative strains was relatively weaker. This distinction was evident from the larger inhibition zones observed for Gram-positive bacteria (ranging from 9.6 to 14.3 mm), indicating a potentially robust inhibitory effect. In contrast, the inhibition zones observed for Gram-negative bacteria were generally smaller (ranging from 7.3 to 9.2 mm), implying a comparatively weaker effect against this bacterial group. The results were like the study conducted by Soyingbe et al. demonstrated the ability of *C. asiatica* extract to inhibit both Gram-positive and Gram-negative bacterial strains [27]. However, our findings specifically highlight the substantial inhibitory effect of the ethanolic extract against *S. mutans* ATCC 25175, as evidenced by an inhibition zone of 14.3 mm. Remarkably, this outcome was like the inhibitory effects achieved by a vancomycin drug. The relevance of this finding lies in its implications for oral health, as *S. mutans* is an important contributor to dental caries and other oral diseases [28, 29]. Additionally, it is worth noting that the choice of solvent used in the extraction process significantly influences the antibacterial activity of the extracts [30].

While our current findings offer valuable insights into the antibacterial potential of the *C. asiatica* ethanolic extracts, further study is necessary to advance our understanding in this area. Additionally, it is crucial to thoroughly evaluate the safety and efficacy of the *C. asiatica* extracts for various therapeutic applications, particularly in the context of mammalian cells and bacterial infection management.

The investigations focus on the isolation and characterization of the bioactive compounds responsible for the observed effects. HPLC-PDA was used to analyze the ethanolic extracts of *C. asiatica* and contributed significant insights into the chemical composition of these extracts. This analytical technique enabled the identification of compounds likely responsible for the observed antibacterial activity. Among the numerous compounds detected in the ethanolic extracts, three principal compounds were identified, such as madecassic acid, madasiatic acid, and asiatic acid [2, 30]. Asiatic acid is a triterpene compound with a chemical structure of “3,3′-methylene-bis(4-hydroxybenzaldehyde),” which is prominently found in *C. asiatica* [15, 24]. Extensive research has associated asiatic acid with various bioactive properties, including antibacterial activity [7, 31]. Similarly, madecassic acid, a terpene compound, presents in the ethanolic extracts of *C. asiatica* leaves [12]. It has also been linked to diverse biological activities [7, 12, 32], including potential antibacterial effects. The present study revealed the presence of madasiatic acid, another bioactive compound detected at a retention time of 7.3 minutes, sharing similarities with asiatic acid. This compound has been reported to exhibit wound healing and neuroprotective effects [6, 21]. The antibacterial activity observed with the ethanolic extracts can likely be attributed to the presence of these bioactive compounds, warranting further investigation into their contributions and potential therapeutic applications.

Exploring the detailed mechanisms underlying the antibacterial effects of these compounds could lead to the development of targeted antibacterial agents or pharmaceutical applications derived from *C. asiatica*. Continued academic and scientific research in this area holds great promise for advancements in antibacterial therapy and the field of natural product pharmacology. Such dental caries, a prevalent oral disease, is primarily attributed to the formation of biofilms, particularly by the bacterium *S. mutans* [26, 28]. This bacterium plays a pivotal role in the development of dental cavities, possessing cariogenic properties [29, 33]. Moreover, this pathogen holds significant relevance to cardiovascular inflammation, as it can potentially enter the bloodstream through inflamed gums or periodontal pockets, leading to life-threatening complications [34]. In light of these factors, our study investigated the inhibitory effects of the *C. asiatica* ethanolic extracts on the biofilm production of *S. mutans* ATCC 25175 as a representative strain, which is closely associated with dental diseases.

This study demonstrated a significant concentration-dependent inhibition of biofilm production by the ethanolic extracts. Notably, noteworthy inhibition of biofilm formation was observed at concentrations of sub-MIC at 1/4 × MIC and 1/2 × MIC of the extracts. Interestingly, the ethanolic extract at 1/2 × MIC exhibited levels of inhibitory effects comparable to that of the standard drug 0.2% chlorhexidine. The results suggest that the ethanolic extracts possess the potential to serve as effective inhibitors of *S. mutans* biofilm formation. Moreover, we hypothesized that the ethanolic extracts might modify the surface properties of *S. mutans* cell surface, potentially affecting their aggregation behavior and biofilm formation [28, 35]. The MATH assay demonstrated that the ethanolic extracts indeed induced significant modifications in the hydrophobicity of the bacterial cells. After exposure to concentrations at sub-MIC of the ethanolic extracts, the hydrophobicity of the bacterial cells was significantly decreased. This observation is crucial as changes in cell surface hydrophobicity can impact the bacterial aggregation and adhesion virulent factors, which are key processes in biofilm formation [35]. The ability of the ethanolic extracts to reduce the hydrophobicity of *S. mutans* cells suggests a mechanism through which these extracts inhibit biofilm formation [29].

Previous studies have extensively elucidated the mechanism by which GtfC catalyzes glucan formation [36]. This process involves the hydrolysis of sucrose, a natural substrate for GtfC, through proton attack. The resulting glycosyl moiety is bound to amino acid residues in subsite-1 of GtfC as an intermediate, while fructose is released from subsite+1 of the enzyme. Critical amino acid residues, namely, Arg475, Asp477, Glu515, His587, Asp588, and Tyr916, play a pivotal role in subsite-1, thereby facilitating the synthesis of glucans. Similarly, amino acid residues Tyr430, Leu433, and Trp517, located at subsite+1, are responsible for recognizing the glucosyl moiety [17, 37]. Furthermore, previous studies have consistently confirmed the pivotal role of Asp588, Tyr517, and Asn481 in catalyzing the hydrolysis of sucrose, which acts as the natural substrate for GtfC. Importantly, any favorable interaction, particularly involving hydrogen bonding, significantly disrupts the native catalytic functions of these residues, ultimately leading to the inhibition of the enzyme [17, 38]. The molecular docking analysis of natural compounds isolated from *C. asiatica,* specifically asiatic acid, madecassic acid, and madasiatic acid, revealed their interactions with multiple amino acid residues in the catalytic site of GtfC, involving hydrogen bonding and hydrophobic interactions. These interactions may impede the utilization of the natural substrate (sucrose) for water-insoluble glucan synthesis. Consequently, asiatic acid, madecassic acid, and madasiatic acid hold significant potential as lead compounds for the development of preventive agents against biofilm formation caused by *S. mutans*.

Moreover, these findings demonstrate the effect of ethanolic extract of *C. asiatica* on NO production in LPS-stimulated RAW 264.7 cells. *C. asiatica* ethanol extract significantly reduced NO production. This reduction is noteworthy, as high NO levels are often associated with inflammation and oxidative stress [39]. Interestingly, the value of NO production in aspirin-treated LPS-stimulated RAW 264.7 cells was not significantly different from that of *C. asiatica* ethanol extract. This suggests that *C. asiatica* ethanol extract was as effective as aspirin in reducing NO levels in these stimulated cells. These results are promising and indicate that *C. asiatica* ethanol extract has the potential to reduce inflammation, which corresponds to the anti-inflammatory of madecassic acid [32, 40] and asiatic acid, the bioactive compounds [13, 15, 24, 41].

## 5. Conclusion

The study suggested that the ethanolic extracts of *C. asiatica* could be used as natural agents against bacterial infections especially *S. mutans* infections, as they exhibited promising antibacterial activities. The modifications of bacterial cell surface were observed after treating the bacterial cells with the ethanolic extracts by decreasing the cell surface hydrophobicity and significantly reducing bacterial biofilm formation within 24 hours. The inhibitory actions on bacterial biofilms were confirmed by a demonstration of 3D structural interactions between the compounds isolated from the *C. asiatic* ethanolic extract and the GtfB amino acids of *S. mutans*. The molecular docking revealed a high docking score of asiatic acid, madecassic acid, and madasiatic acid isolated from the plant ethanolic extract. The activity was close to those of chlorhexidine, suggesting that it was an *in vitro* biofilm inhibitor in this study. Moreover, the ethanolic extract demonstrated significant reducing NO production in LPS-stimulated RAW 264.7 cells. The anti-inflammatory activity could be compared to the action of the aspirin drug. The findings indicated the applications of the *C. asiatica* ethanolic extract as the alternative anti-*S. mutans* agent and could be used for further formulation for the treatment and prevention of dental diseases and inflammatory injury in the oral cavity.

## Figures and Tables

**Table 1:** Inhibition zone of the *C. asiatica* extracts at the concentration of 25 mg per disk and standard antibiotics against pathogenic bacteria on agar culture.

Bacterial strain	Diameter of inhibitory zone (mm)			
	Ethanolic extract of *C. asiatica* (25 mg)	Aqueous extract of *C. asiatica* (25 mg)	Vancomycin (30 *μ*g)	Ciprofloxacin (5 *μ*g)
*Staphylococcus aureus* ATCC 25923	9.6 ± 0.5	6.5 ± 0.5	19.5 ± 0.5	NA
*Staphylococcus epidermidis* ATCC 35984	10.5 ± 0.0	7.0 ± 1.0	24.2 ± 0.5	NA
*Streptococcus mutans* ATCC 25175	14.3 ± 1.0	8.4 ± 0.5	17.5 ± 0.5	NA
*Escherichia coli* ATCC 25922	9.2 ± 0.5	6.8 ± 0.5	NA	21.5 ± 0.0
*Klebsiella pneumoniae* ATCC 700603	7.5 ± 0.5	6.2 ± 1.0	NA	19.0 ± 1.0
*Acinetobacter baumannii* ATCC 17978	8.5 ± 1.0	6.5 ± 1.0	NA	21.2 ± 0.5
*Pseudomonas aeruginosa* ATCC 27853	7.3 ± 0.5	6.7 ± 0.5	NA	18.5 ± 1.0

NA: not applicable.

**Table 2:** MICs and MBCs of *C. asiatica* extracts against pathogenic bacterial strains.

Bacterial strain	MIC/MBC of antibacterial agents (mg/mL)			
	Ethanolic extract of *C. asiatica*	Aqueous extract of *C. asiatica*	Vancomycin	Ciprofloxacin
*Staphylococcus aureus* ATCC 25923	2.048/4.096	32.768/>65.536	0.001	NA
*Staphylococcus epidermidis* ATCC 35984	1.024/2.048	32.768/>65.536	0.0005	NA
*Streptococcus mutans* ATCC 25175	1.024/2.048	16.384/32.768	0.00025	NA
*Escherichia coli* ATCC 25922	8.192/16.384	>32.768/>65.536	NA	0.0005
*Klebsiella pneumoniae* ATCC 700603	16.384/32.768	>32.768/>65.536	NA	0.00025
*Acinetobacter baumannii* ATCC 17978	16.384/32.768	>32.768/>65.536	NA	0.0005
*Pseudomonas aeruginosa* ATCC 27853	16.384/32.768	>32.768/>65.536	NA	0.00025

NA: not applicable.

**Table 3:** The principal compounds isolated from the ethanolic extract of *C. asiatica* and qualified by high-performance liquid chromatography coupled with a photodiode array detector (HPLC-PDA).

Peak	Retention time (RT) (min)	Name of the compound	Molecular formula	Molecular weight (g/mol)
1	6.7	Madecassic acid	C_30_H_48_O_6_	504.7
2	7.3	Madasiatic acid	C_30_H_48_O_5_	488.7
3	7.7	Asiatic acid	C_30_H_48_O_5_	488.7

**Table 4:** The binding affinity and interacting amino acid residues of the compounds isolated from *C. asiatica* ethanolic extract with GtfC from *S. mutans* (PDB: 3AIC).

Compound	Binding affinity (kcal/mol)	Hydrogen bond interaction		Hydrophobic interaction	
		Number of interactions	Amino acid residues	Number of interactions	Amino acid residues
Alpha-acarbose (a cocrystallized ligand and known inhibitor) (C_25_H_43_NO_18_)	−8.2	15	**T** **Y** **R**430, **ARG**475, **ASN**481^**a**^, **GLU**515, ARG540^a^, **HIS**587, **ASP**588^**a**^, ASP593, TYR610, ASP909, GLN960^a^	1	**LEU433**
Chlorhexidine (a positive control) (C_22_H_30_Cl_2_N_10_)	−8.4	4	**T** **Y** **R**430^**a**^, ALA478, **ASN**481	8	LEU382, **TYR**430, **LEU**433, LEU434, ASP480, **TRP**517^**a**^, PHE907
Asiatic acid (C_30_H_48_O_5_)	−8.8	4	**H** **I** **S**587, **ASP**588^**a**^, GLN592	1	LEU434
Madecassic acid (C_30_H_48_O_6_)	−10.0	9	**TYR430**, **ARG475**, **ASP477**, **HIS587**, **ASP588**, GLN592, ASN862, ASP909, ASN914	3	**LEU433**, LEU434, **TRP517**
Madasiatic acid (C_30_H_48_O_5_)	−10.0	5	**A** **S** **P**477, **HIS**587, GLN592^a^, ASN862	5	**L** **E** **U**433, LEU434, **TRP**517, **TYR**916^**a**^

^a^Two interaction with amino acid residues. Amino acid residues in the active site of GtfC of *S. mutans*, as shown in bold.

## Data Availability

The data used to support the conclusions drawn in this research are integrated into the article.
